# A collaborative privacy-preserving approach for passenger demand forecasting of autonomous taxis empowered by federated learning in smart cities

**DOI:** 10.1038/s41598-024-52181-6

**Published:** 2024-01-24

**Authors:** Adeel Munawar, Mongkut Piantanakulchai

**Affiliations:** Sirindhorn International Institute of Technology, Thammasat University, Pathum Thani, 12120 Thailand

**Keywords:** Engineering, Civil engineering

## Abstract

The concept of Autonomous Taxis (ATs) has witnessed a remarkable surge in popularity in recent years, paving the way toward future smart cities. However, accurately forecasting passenger demand for ATs remains a significant challenge. Traditional approaches for passenger demand forecasting often rely on centralized data collection and analysis, which can raise privacy concerns and incur high communication costs. To address these challenges, We propose a collaborative model using Federated Learning (FL) for passenger demand forecasting in smart city transportation systems. Our proposed approach enables ATs in different regions of the smart city to collaboratively learn and improve their demand forecasting models through FL while preserving the privacy of passenger data. We use several backpropagation neural networks as local models for collaborating to train the global model without directly sharing their data. The local model shares only the model updates with a global model that aggregates them, which is then sent back to local models to improve them. Our collaborative approach reduces privacy concerns and communication costs by facilitating learning from each other’s data without direct data sharing. We evaluate our approach using a real-world dataset of over 4500 taxis in Bangkok, Thailand. By utilizing MATLAB2022b, the proposed approach is compared with popular baseline methods and existing research on taxi demand forecasting systems. Results demonstrate that our proposed approach outperforms in passenger demand forecasting, surpassing existing methods in terms of model accuracy, privacy preservation, and performance metrics such as Root Mean Square Error (RMSE), Mean Absolute Error (MAE) and R-squared ($R^2$). Furthermore, our approach exhibits improved performance over time through the collaborative learning process as more data becomes available.

## Introduction

Smart cities are indispensable for providing sustainable, efficient, and integrated urban environments that improve the quality of life for their residents while driving economic development^1^. The fundamental objective of smart cities is to optimize available resource utilization for the benefit of all stakeholders (e.g., residents, service providers, governments, and businesses)^2–4^. One such challenge is the integration of Autonomous Taxis (ATs) into smart cities, where they can play a crucial role in addressing the pressing issues of rapid population growth and urbanization. By optimizing energy consumption, reducing traffic congestion, and minimizing passenger and taxi waiting times, it emerged as a promising solution for sustainable and efficient transportation in smart cities^5,6^.

Taxis are vital to urban public transport systems, providing convenient and personalized services that greatly impact people’s lives in cities. However, driver profitability is a concern^7^, as their earnings are directly linked to driving efficiency^8^. Literature shows that reducing cruising distance is key to increasing profits, but taxis waste a significant amount of time cruising the roads in search of passengers, resulting in decreased efficiency^9^. Despite the potential offered by GPS tracking technology to optimize routes, accurately predicting passenger demand and pickup probabilities remains challenging due to dynamic factors (e.g., time and location)^10^. As we move towards integrating Autonomous Vehicles (AVs) in Intelligent Transportation Systems (ITS), it becomes crucial to accurately forecast passenger demand for ATs in urban areas, particularly in smart cities. Accurate passenger demand forecasting is essential for minimizing passenger waiting time and optimizing the cruising time of ATs in metropolitan areas^11^. Improving these aspects enhances the passenger experience, reduces energy consumption, and increases overall transportation system efficiency in smart cities. In the foreseeable future, taxi companies like Uber and Lyft may actively operate combined fleets of electric and AVs to offer passenger pickup and transportation services in smart cities^12,13^. The field of AVs is rapidly growing, with companies such as CRUISE in California already providing fully autonomous driverless taxi services under specific conditions^14^. Although smart cities adopt ITS and AVs, the key operational challenge is to accurately match taxi supply with passenger demand to minimize waiting times and optimize the utilization of ATs. Machine learning (ML) has made significant contributions to the development of forecasting models, providing improved performance and cost-effective solutions in the AVs industry^15–17^. Passenger demand forecasting is an integral component of the ATs service, as it enables the efficient allocation of taxis to passengers. But ML-based solutions require a massive amount of data for training, which can be challenging to obtain due to data privacy and security concerns. Data is the most valuable asset for every organization, but authorities are reluctant to share it due to data protection laws and concerns about security and privacy^18,19^. Data sharing raises considerable privacy concerns due to its potential to reveal highly personal information. This includes sensitive details such as individuals’ exact locations, movement patterns, and even their religious, political, or sexual convictions. This information can be inferred by predicting Points of Interest (POI) using mapping data and coordinates, amplifying the need for caution when sharing such data. Researchers have developed novel ML approaches^11,20–22^ to address this issue and hybridized existing ones to create efficient prediction models. It’s important to note that these ML-based solutions used in the transportation industry tend to be centralized, requiring collecting and storing sensitive passenger data in a centralized location, raising the possibility of network latency, connection delays, data theft, and other privacy or security concerns. Also, sending large amounts of passenger data over long distances can be prohibitively expensive for any transportation service provider. In the ATs business, privacy issues can be more serious than in other businesses. To address these challenges, we need an alternative and reliable method for predicting passenger demand for ATs while maintaining passenger privacy, network latency, and security-related issues so passengers’ trust can be preserved by all means possible.

**Figure 1:**
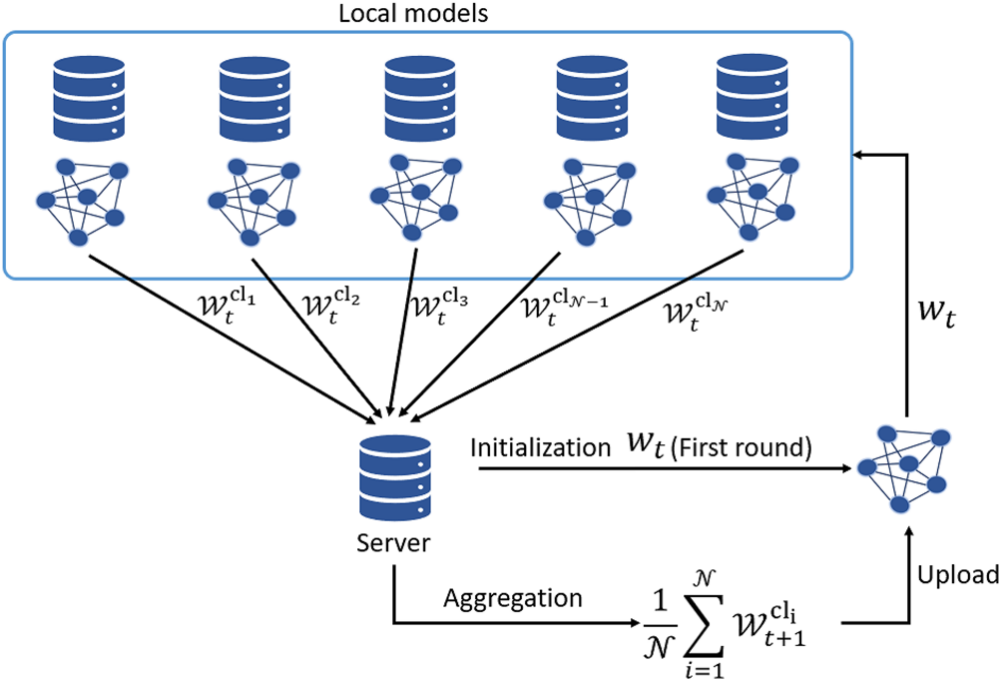
Framework of the collaborative approach.

Federated Learning (FL) is an alternative ML approach that enables clients to process data locally and independently train their own models while maintaining the privacy and security of their data. FL reduces communication overhead and eliminates network issues, making it an ideal solution for secure and privacy-protected prediction models for ATs^20^. FL-based models provide a decentralized learning approach, which is highly beneficial for the ATs industry. ATs companies can train accurate and secure models that can efficiently allocate taxis to passengers while protecting passenger privacy and safety. FL can revolutionize the AT industry by allowing companies to provide more efficient, safe, and reliable services^20,21, 23^. FL is a promising approach for collaborative ML that prioritizes data privacy. It enables multiple entities or devices to train a global model without directly sharing local data. In the context of ATs, FL enables collaboration and the development of passenger demand forecasting models in different regions without directly sharing passenger data. Figure 1 shows the basic framework of the FL-based collaborative approach.

Where the parameter of the global model is represented by *w*, while the total number of clients is denoted by ${\mathscr {N}}$, and the communication round in the FL process is represented by *t*.

In this paper, we propose a collaborative model based on the FL approach for passenger demand forecasting for ATs within smart cities. Our proposed approach empowers ATs to learn from their local data without compromising privacy or incurring high communication costs. To evaluate the effectiveness of our proposed approach, we conduct experiments by utilizing real-time historical data from more than 4500 taxis in Bangkok, Thailand, collected through Internet of Things (IoT) devices installed in taxis. Our experimental results of the proposed model are compared with existing studies and state-of-the-art approaches related to demand forecasting. Simulation results demonstrate that our approach achieves a high accuracy rate in passenger demand forecasting, surpassing existing methods in terms of model accuracy, privacy preservation, and performance metrics such as Root Mean Square Error (RMSE), Mean Absolute Error (MAE), and R-squared ($R^2$).

The remainder of this paper is organized as follows: In “Literature review” section, we review related work in passenger demand forecasting and FL, discussing the existing approaches. “Methodology” section describes the proposed collaborative approach based on FL, providing a detailed description of its key components with a detailed mathematical description. “Proposed model” section shows the simulation results and performance evaluation, demonstrating the effectiveness of our proposed approach and comparing it with existing studies and state-of-the-art approaches related to passenger demand forecasting. “Results and discussion” section draws attention to our findings regarding the significant contribution that our proposed approach makes to enhancing efficient and sustainable modes of transportation within smart cities. Our insights lead us to identify potential future research directions to examine FL techniques and the potential for further development in ITS.

## Literature review

In urban areas, taxi services have been a popular mode of public transportation for decades. However, with the rapid development of information and communication technology and the emergence of smart cities, ATs have become the new frontier in the transportation industry. Our study focuses on passenger demand forecasting for ATs in smart cities, aiming to optimize pickup probability and improve passenger service rates. The main challenge is accurately predicting taxi requests at specific locations at a given timestamp. The efficient allocation of taxis and their optimal utilization depends on forecasting the probability of pickups in different areas. Our research aims to contribute to developing advanced techniques for enhancing passenger demand forecasting, leading to improved service quality and operational efficiency in the ATs industry.

Several studies have been conducted on passenger demand forecasting, particularly regarding taxi pickup, traffic congestion, and route management. An Auto-Regressive Integrated Moving Average (ARIMA) method is one of these studies that use the Box–Jenkins method. Although it considers past patterns within a given period to predict future outcomes, it is generally considered better for long-term rather than short-term predictions^20,23, 24^. Qu et al.^21^ identify hotspots and estimate pickup probability using the Kalman filtering method. They proposed an iterative local search strategy to maximize the pickup probability for drivers. In another study, Qu et al.^22^ used a clustering approach to determine popular pickup locations from a large dataset and then recommend these locations to taxi drivers. Naji et al.^25^ proposed a deep learning model for taxi demand forecasting, utilizing Generative Adversarial Networks (GANs) with multi-source data. The model, incorporating recurrent and conventional network components, is evaluated on real-world datasets from Wuhan, China. The model exhibits exceptional performance through a comparative examination, particularly in the Guanggu area, with an RMSE of 9.78 and MAE of 16.75, superior to the efficacy of alternative baseline methods. Markou et al.^26^ proposed a real-time taxi demand prediction model using ML and web data. Their models significantly reduce forecast errors. The dataset utilized is from the NYC Taxi and Limousine Commission (TLC). The most efficient model attains MAE of 8.301 and RMSE of 14.932. In study^27^, Zhao et al. introduced a unified framework that leverages multi-source data for enhanced accuracy. The study proposes and evaluates two deep models, USTN and STIMN, using datasets from taxis and Uber in New York City. The STIMN model consistently outperforms in various scenarios, achieving the best prediction with an RMSE of 14.04 and an MAE of 8.32. An other Study^28^, proposed a Multi-Attribute Spatial–Temporal Graph Convolutional Network (MASTGCN) for taxi demand forecasting. The model incorporates historical cab inflows and spatial dependencies, utilizing multiple attributes for accuracy. MASTGCN’s four components capture both temporal and spatial dependencies, exhibiting superior performance. The model’s best accuracy was observed in Brooklyn data, with an RMSE of 9.32 and MAE of 6.65. In Ref.^29^, Fiosina proposed a FL approach for traffic prediction that allows the processing of distributed data while ensuring privacy preservation. Another study^30^ proposed FedGRU for predicting traffic flow using FL to protect privacy. FedGRU operates by employing a secure parameter aggregation mechanism, allowing for the training of a global model in a distributed manner without directly accessing the distributed organizational data. In a study on smart cities, the authors of study^31^ discuss the challenges and opportunities of FL. They highlighted security, privacy, and data collection challenges. In the study by Imteaj and Amini^32^, focused on data collection from smartphones’ built-in sensors in smart city infrastructure for transportation, health, and emergency services. Silva et al.^33^ proposed FL for real-time traffic forecasting using roadside units and suggested exploring different models. Lonare et al.^34^ presented an aggregated approach to predict vehicle traffic, utilizing decentralized local data securely shared with the server and training with spatial parameters. In the literature mentioned above, various authors employ diverse methodologies to enhance the results of demand forecasting. Table 1. Compares several existing studies, highlighting their methodologies, datasets, and accuracy levels.

**Table 1:** Comparison of previous studies based on accuracy and dataset.

Study	Model	Accuracy		Dataset
		RMSE	MAE	
Naji et al.^25^	GANs	9.78	16.75	Real-world datasets of Wuhan, China
Markou et al.^26^	ML with linear regression and Gaussian processes	14.94	8.31	TLC and NYC Taxi
Zhao et al.^27^	USTN and STIMN	14.04	8.32	Taxi and Uber datasets of NYC, USA
Xu et al.^28^	MASTGCN	9.32	6.65	Brooklyn, Manhattan, and Haikou-Didi-Taxi

Our study presents several significant contributions to the emerging field of demand forecasting for ATs in smart cities. We propose a collaborative model based on FL, enabling ATs in different regions to collectively enhance their demand forecasting models. To facilitate this collaboration, we introduce an innovative aggregation protocol that efficiently combines model updates without compromising data confidentiality. Through extensive experiments on real-world trip data from over 4500 taxis in Bangkok, we demonstrate our collaborative approach’s superiority over individual learning. Our approach achieves significantly improved forecasting accuracy by leveraging collective knowledge. This collaborative approach ensures the preservation of passenger data privacy, a critical concern in today’s data-driven world.

Our work distinguishes itself from the existing literature by seamlessly integrating a collaborative ML approach, particularly FL, into the domain of passenger demand forecasting for smart city transportation systems. While prior research has primarily focused on standalone forecasting models, our approach capitalizes on a collaborative approach to enhance accuracy and privacy protection and reduce communication costs. This novel approach aligns with the evolving landscape of smart cities, where sustainable and efficient transportation systems are needed.

Our motivation is rooted in the pressing need for sustainable and efficient transportation systems in smart cities. By applying collaborative learning techniques, we improve the accuracy of passenger demand forecasts and safeguard individual privacy. Our research brings innovation by demonstrating that advanced technologies can be coupled to enhance passenger experiences, reduce communication costs, and contribute to developing a more sustainable future for smart cities.

### Federated learning

FL is a relatively new approach to ML that originated in the early 2010s. It was introduced by Google researchers in 2016 as a privacy-preserving alternative to centralized ML approaches, where devices collaborate to train a joint model without sharing raw data^35^. It is widely adopted across domains, enabling efficient ML while ensuring data privacy and legal compliance among multiple parties or computing nodes. Some examples of these domains include virtual keyboard prediction^36^, smart retail^37^, network safety^38^ and vehicle-to-vehicle communication^39^. ATs face unresolved data privacy, security, and availability issues. Developing a model to accurately predict passenger demand while protecting data privacy, security, and availability is critical.

The main idea behind FL revolves around enabling multiple data owners or clients to collaboratively train a shared ML or deep learning model without revealing their private data, addressing privacy concerns^35,40, 41^. Clients and an FL server are the main components of the FL framework. Each client uses their private data to train their local model and sends the accurately computed parameters to the FL server. Instead of transmitting data over a network, FL aggregates small-scale local models trained from onsite data on a central server to create a global data model. FL consists of three main components that play an important role in the overall process^42^.

*Initialization phase* In this phase, the server sets data requirements for each client and configures the model’s hyperparameters. Once the model is initialized, it is distributed to the active clients for local training.

*Local training phase* During this phase, clients start training using their local data. They iteratively optimize their parameters based on a predefined cost function, leveraging their computational resources while ensuring data privacy and security. Clients continuously learn from their data, updating their model parameters accordingly and send the updated parameter to the server after each round of local training.

*Server upgrade phase* This phase involves integrating the clients’ local parameters through the aggregation procedure, computing the updated global model parameters, and optimizing the global cost function. Clients receive these updated global parameters iteratively until the global model achieves the desired performance or convergence. These three phases collaboratively support shared learning while protecting data privacy. They enable the creation of a collaborative model that integrates knowledge from decentralized clients. Table 2 provides an overview and comparison of the FL to various learning techniques^43^.

**Table 2:** Comparison of learning techniques^43^.

Techniques	Description	Communication costs	Distribution of data	Efficiency	Privacy
Datacenter	Distributing the data across several servers for parallel processing	High	Multi-center	Moderate	Low
Centralize	Aggregate the data in one place for processing by a centralized ML model	High	Central	High	Low
MapReduce	Moving the processing unit to the data allows the task to be split among clients and handled concurrently	Low	Distributed	Moderate	Low
Shared ML	Promotes collaborative sharing of ML models, data, and resources, enhancing efficiency	Moderate/high	Distributed	High	Moderate
Federated	Preserving approach where multiple devices train a shared model by exchanging model updates without sharing raw data	Low	Distributed	Moderate	High

## Methodology

This section presents the proposed collaborative approach based on FL and provides a comprehensive description with a detailed mathematical description of the key components of the proposed approach.

### Dataset description

**Figure 2:**
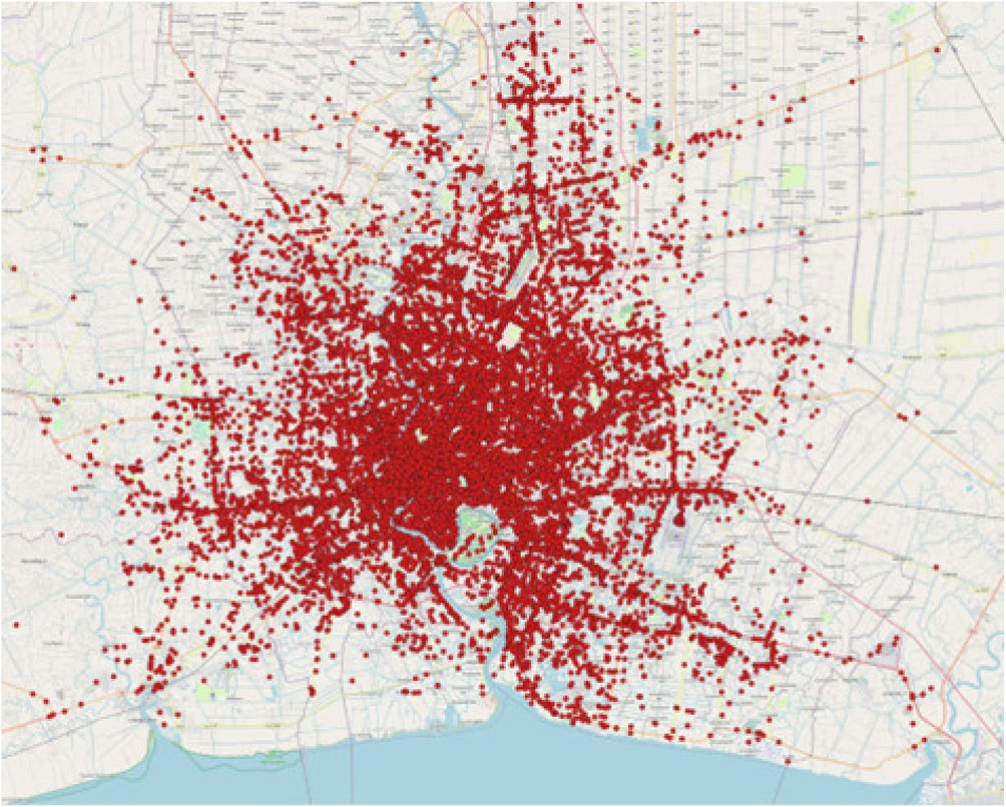
Visualization of the single-day pickups.

The data set used in the study consists of historical raw vehicles and mobile probes data from Thailand^44^, offering extensive spatial and temporal coverage of Bangkok. The data set includes information on taxi GPS records, such as unique taxi IDs, location coordinates, GPS accuracy, timestamps, taxi speed, direction, taxi meter status, and record update frequency. The data set spans four months, from September to December 2021, three months for training and one month for testing, with 247822541 records from 4781 taxis. Figure 2 shows the visualization of the single-day pickup locations in Bangkok, Thailand.

### Preprocessing

**Figure 3:**
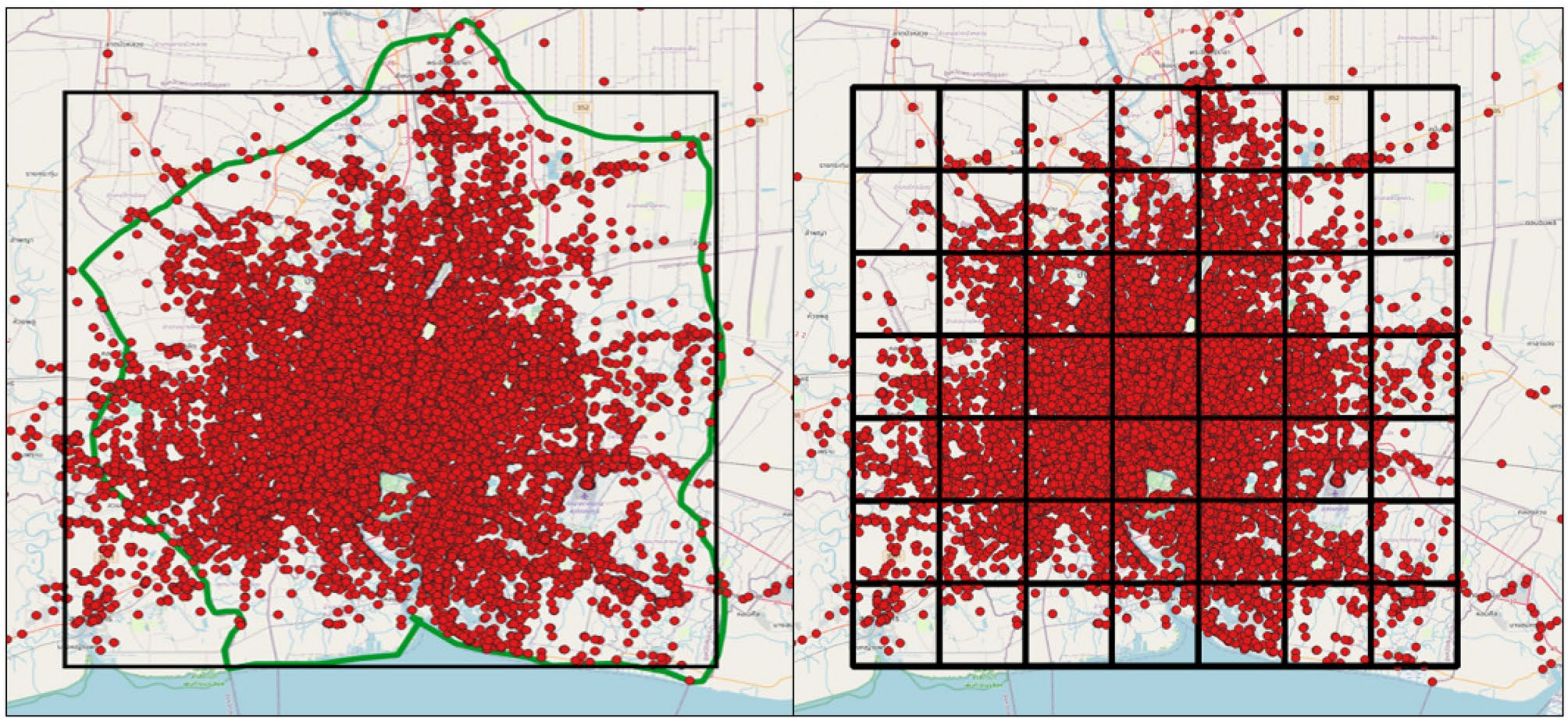
AOI, boundary limit, pickup points (left) and grid distribution (right).

Data preprocessing involves transforming and encoding data to enable easy interpretation by ML algorithms. In the context of taxi demand forecasting, we analyze consecutive pickup and drop-off events from taxi GPS trajectory data. We assume the trajectory data represents all taxi movements and activities. To reduce computational load, we limited our focus to the Area of Interest (AOI) in Bangkok, Thailand. We eliminated outliers outside the defined boundary box. We also filtered anomalies, such as missing and duplicate records, using R language and R Studio^45^. Entries with incorrect coordinates were removed. Overall, 22% of records were eliminated. The AOI, boundary limit, and pickup points are shown on the left side of Fig. 3. Pick-up and drop-off points of passengers were identified based on changes in the taximeter feature of our data set. When the meter changed from 0 to 1, it marked the source location, and when it changed from 1 to 0, it indicated the destination. Sorting the data by time allowed us to identify the transition between taxi occupancy and idleness. This study focused on a specific region of Thailand between latitude $-\,13.50099^{\circ }$ to 14.10965$^{\circ }$ and longitude 100.24778$^{\circ }$–$100.87591^{\circ }$. This region encompassed various locations such as shops, train stations, neighborhoods, and tourist attractions, making it representative of the overall taxi situation in the city. Using the equal-size grid approach, we divided the area into 7 $\times$ 7 grids, as shown on the right side of Fig. 3, each grid covering 10 km $\times$ 10 km. This division facilitated forecasting and scheduling by assigning a unique ID to each record based on its location within the grid.

**Figure 4:**
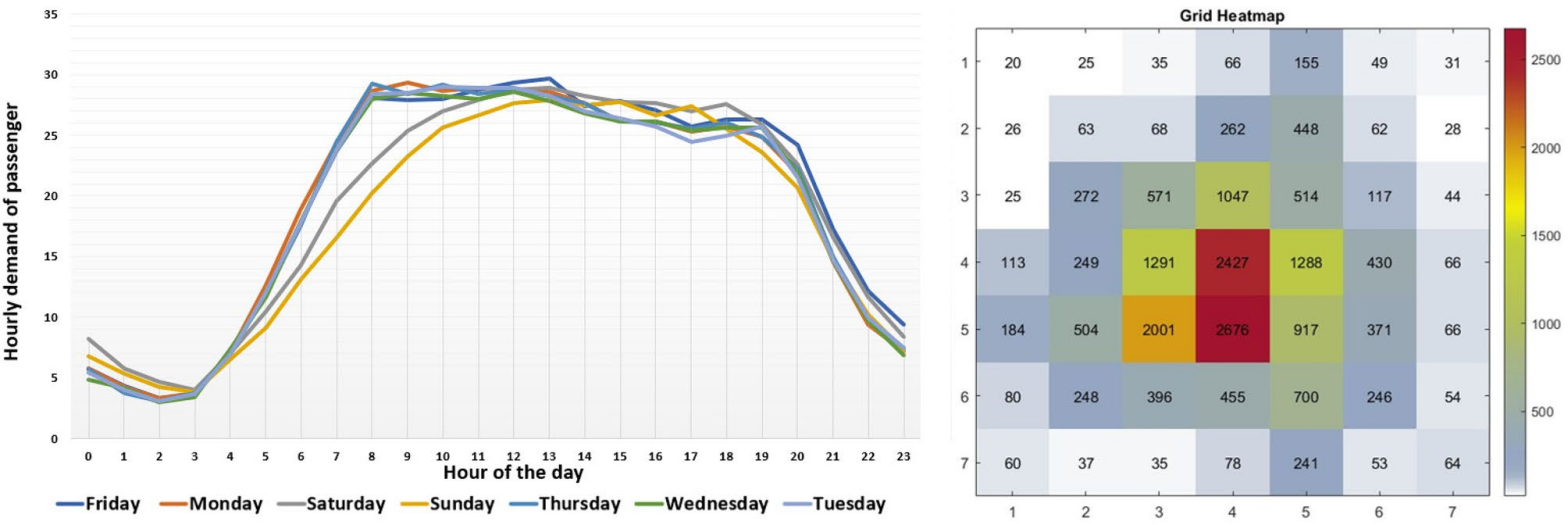
Day-wise passenger pickup trend for 24-hour time slots (left) and comparison heatmap of average demand across grids (right).

Passenger pickup frequency is used as a measure of passenger demand. We collected and aggregated hourly pickup frequency data for each grid to analyze demand patterns. This provided us with 24-hour intervals of taxi demand data for each grid. Figure 4 shows the time trend of passenger demand at different time slots throughout the week and a heatmap comparing demand across grids within a selected AOI.

## Proposed model

**Figure 5:**
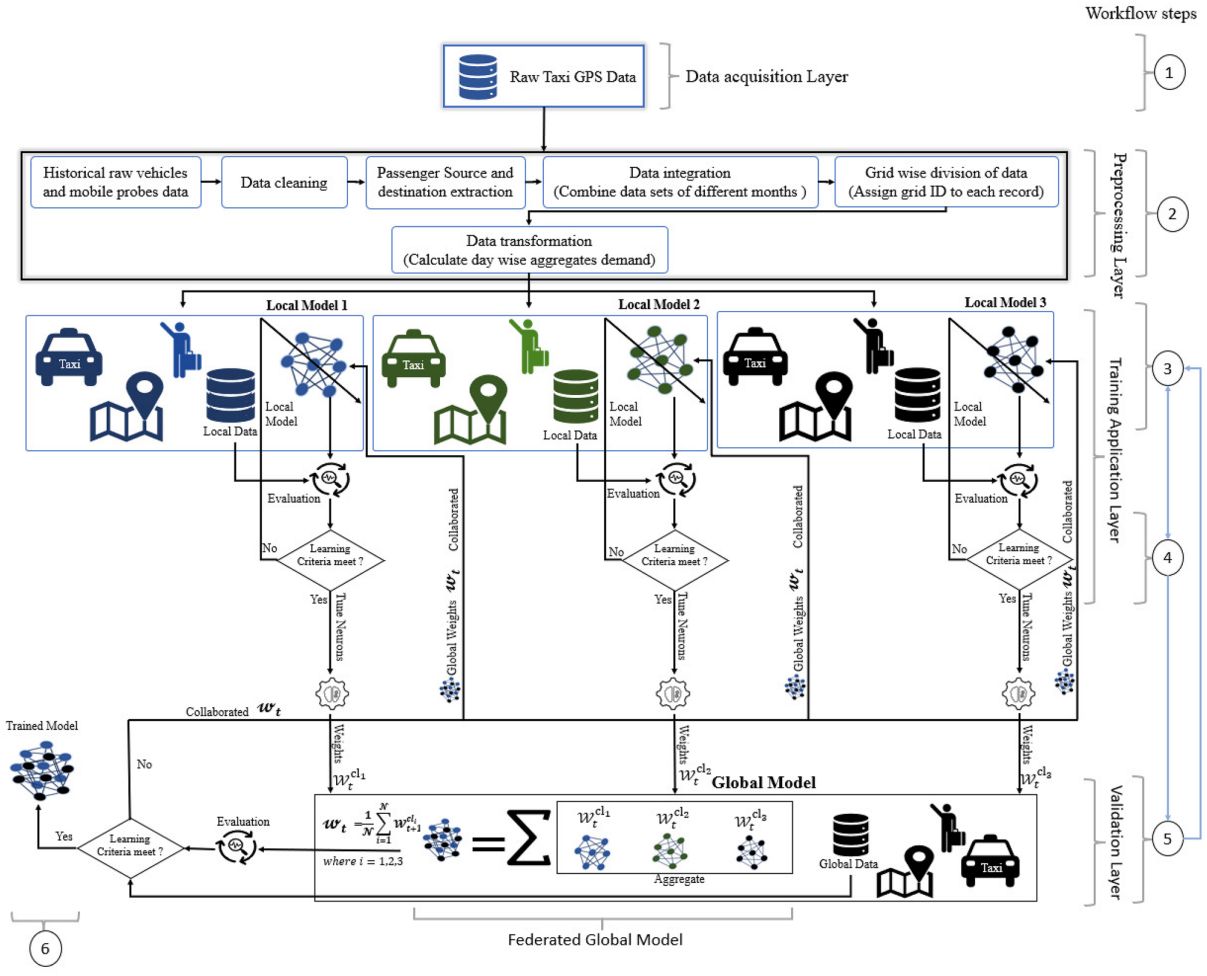
Proposed collaborative model framework.

This section provides a concise description of a collaborative model for passenger demand forecasting for ATs using FL, along with a comprehensive explanation of the ANN as the fundamental component of the model. The model aims to accurately predict demand while addressing privacy concerns and reducing communication costs associated with centralized data collection and analysis. As shown in Fig. 5, the model consists of several interconnected phases, each with a distinct purpose. Additional Fig. 5 also includes the workflow steps, providing a visual representation of the entire process for better understanding the flow. First, the acquisition layer collects the required data using IoT devices such as a taxi GPS device, which senses the data wirelessly (Step 1). After data collection, the preprocessing phase comes into play. The raw data undergoes preprocessing techniques to eliminate noise and enhance the quality of the data set. This step ensures that subsequent modeling and analysis are based on clean and reliable data (Step 2).

The next phase involves training and retraining local models, where each model acts as a client and trains itself using a Bayesian backpropagation neural network. This training is performed using the available taxi data that is randomly distributed among them (Step 3). Each model incorporates its individualized learning criteria and adjusts its parameters to enhance overall performance and accuracy (Step 4). This localized training approach enables ATs in various smart city regions to learn and participate in training global demand forecasting models collaboratively. The FL approach is used to preserve passenger privacy and reduce communication costs. Local models share only their model updates rather than raw passenger data with the global model. In our simulation, three clients were selected, and each client randomly selected a set of 1500 data samples. The model framework is illustrated in Fig. 5. Aggregation of updates is achieved through a cloud layer, where model updates are aggregated and distributed to local models. The cloud layer facilitates the exchange of average weights between the global model and local models, enabling collaborative learning (Step 5). The iterative process continues for 1500 epochs, converging upon meeting the model’s stopping criteria. The convergent state is distinguished by identifying the optimal result through the evaluation of test data in each epoch, signifying the achievement of the most favourable result (Step 6). After reaching the threshold, the model weights are transferred to the private edge cloud for optimization, enhancing performance, and improving predictions of passenger demand in a smart city environment.

**Figure 6:**
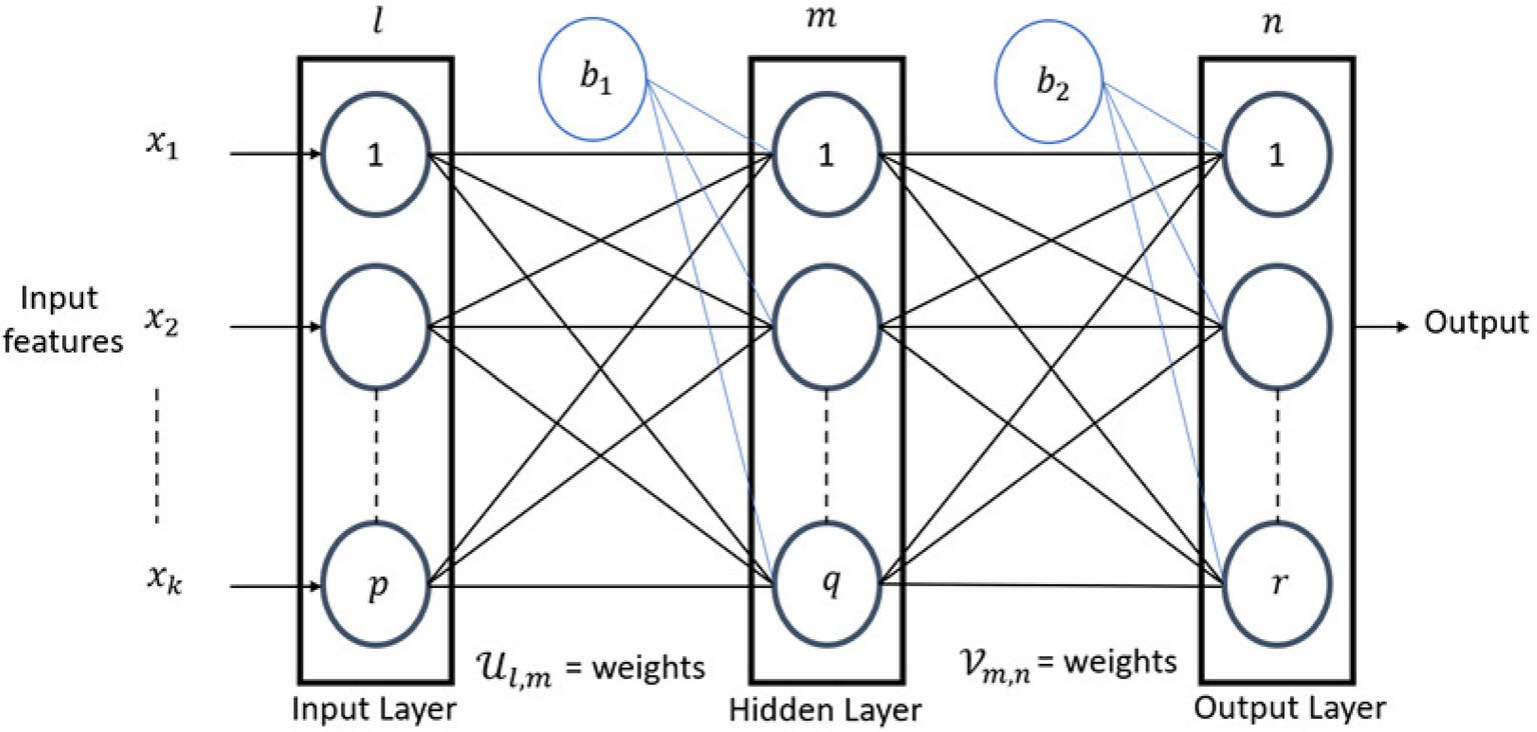
Block diagram of Artificial Neural Network.

In the model architecture, each local model represents an AT or a group of taxis in a specific region. These local models, labelled as model 1, model 2, and so on up to model *N*, contribute to the formation of the global model stored in private edge clouds. This collaborative approach allows the model to utilize data from different sources and improve accuracy, taking into account the diverse nature of the smart city environment. The collaborative approach utilizes Artificial Neural Networks (ANNs) architecture consisting of input, hidden, and output layers. This hierarchical arrangement is inspired by the functioning of human neurons, where each layer performs specific functions, as shown in Fig. 6. The input features are denoted by $\ x_1{,\ x}_{2,\ldots }x_k$. Each circle inside the layer represents a neuron, and the element indices in each layer are represented by *l*, *m*, and *n*.

The weight ${\mathscr {U}}_{l,m}$ connects the input and hidden layers, while the weight ${\mathscr {V}}_{m,n}$ connects the hidden and output layers in a neural network. These weights play a crucial role in determining how information flows, and decisions are made within the network. The dimensions of each layer are defined as follows: *n* represents the total number of elements in the input layer, m represents the total number of elements in the hidden layer, and p represents the total number of features in the output layer. Additionally, Bias terms $b_1$ and $b_2$ are added to each layer, improving the model’s flexibility and performance. The collaborative model includes neural networks with input, hidden, and output layers for each client. It employs the backpropagation algorithm in multiple phases: weight initialization, feedforward, error backpropagation, and weight and bias updating. The hidden layer neurons utilize the Sigmoid activation function.

The output of each neuron in the hidden layer, denoted as ${\mathscr {H}}_m^{{cl}_i}$, for $i\mathrm{^{th}}$ client ${cl}_i$ is calculated using Eq. (1). It is obtained by multiplying the input features with their corresponding weights and adding a bias term, $b_1$^46,47^.

1 $$\begin{aligned} {\mathscr {H}}_m^{{cl}_i}=\frac{1}{1+e^{-\left( b_1+\sum _{l=1}^{p} {{\mathscr {U}}_{m,l}^{{cl}_i}x_i}\right) }},\quad \text {where } 1\le m\le q \end{aligned}$$

where ${x}_i$ refers to the input data, $b_1$ denotes the bias term, *p* represents the total count of input neurons, and *m* corresponds to the total number of neurons in the hidden layer. Similarly, in Eq. (2), the output at the $n\mathrm{^{th}}$ neuron in the output layer is denoted by ${\mathscr {O}}_n^{{cl}_i}$.

2 $$\begin{aligned} {\mathscr {O}}_n^{{cl}_i}=\frac{1}{1+e^{-\left( b_2+\sum _{m=1}^{q} {{\mathscr {V}}_{m,l\ }^{{cl}_i}{\times \ {\mathscr {H}}}_m^{{cl}_i}} \right) }}\ where\ 1\le n\le r \end{aligned}$$

The actual and estimated output error is determined by computing the difference using Eq. (3). Where ${{\mathscr {E}}r}^{{cl}_i}$ denotes the error of the $i\mathrm{^{th}}$ client ${\ cl}_i$, ${\mathfrak {a}o}_n^{{cl}_i}$ represents the actual output, and ${\mathscr {O}}_n^{{cl}_i}$ represents the estimated output at the output layer.

3 $$\begin{aligned} {{\mathscr {E}}r}^{{cl}_i}=\frac{1}{2}\sum _{n=1}^{r} \left( {\mathfrak {a}o}_n^{{cl}_i}-{\mathscr {O}}_n^{{cl}_i}\right) ^2 \end{aligned}$$

The weights that connect the layers are modified to approach an optimal value that reduces the error, as defined in Eq. (3), using the forward and backward propagation approach. To minimize the error, the adjustment of weights between the output layer and hidden layer can be determined by calculating the derivative of the error function with respect to the weight changes, as illustrated in Eq. (4). In this equation, $\Delta {\mathscr {V}}^{{cl}_i}$ represents the weight changes of the ${i}\mathrm{^{th}}$ client at the output layer.

4 $$\begin{aligned} \Delta {\mathscr {V}}^{{cl}_i} = -\frac{\partial {\mathscr {E}}^{\textrm{r}}_{{cl}_i}}{\partial {\mathscr {V}}^{{cl}_i}} \end{aligned}$$

Similarly, the adjustment of weights connecting the hidden layers to the input layer can be computed using Eq. (5). In this equation, $\Delta {\mathscr {U}}^{{cl}_i}$ shows the weight changes of the hidden layer for the ${i}\mathrm{^{th}}$ client.

5 $$\begin{aligned} \Delta {\mathscr {U}}^{{cl}_i} = -\frac{\partial {\mathscr {E}}^{\textrm{r}}_{{cl}_i}}{\partial {\mathscr {U}}^{{cl}_i}} \end{aligned}$$

By employing Eqs. (4) and (5), the relationship between the change in weights can be represented as Eqs. (6) and (7). In these equations, $\Delta {\mathscr {U}}_{l,m}^{{cl}_i}$ represents the weight changes between the hidden and input layer neurons while $\Delta {\mathscr {V}}_{m,n}^{{cl}_i}$ shows the weight changes for each client ${cl}_i$ at each $m\mathrm{^{th}}$ neuron in the hidden layer connected with $n\mathrm{^{th}}$ neuron in the output layer. The equations can be written as follows^47^:

6 $$\begin{aligned}&\Delta {\mathscr {V}}_{m,n}^{{cl}_i} = -\text {const} \frac{\partial {\mathscr {E}}^{\textrm{r}}_{{cl}_i}}{\partial {\mathscr {V}}_{m,n}^{{cl}_i}} \end{aligned}$$

7 $$\begin{aligned}&\Delta {\mathscr {U}}_{l,m}^{{cl}_i} = -\text {const} \frac{\partial {\mathscr {E}}^{\textrm{r}}_{{cl}_i}}{\partial {\mathscr {U}}_{l,m}^{{cl}_i}} \end{aligned}$$

In order to compute $\Delta {\mathscr {V}}$, the chain rule is utilized, as the error arises from the disparity between the output layer and the hidden layer. After using the Chain rule technique above^47^, Eq. (8) can be written as;

8 $$\begin{aligned} \Delta {\mathscr {V}}_{m,n}^{{cl}_i} = -\text {const} \frac{\partial {\mathscr {E}}^{\textrm{r}}_{{cl}_i}}{\partial {\mathscr {O}}_n^{{{cl}_i}}} \times \frac{\partial {\mathscr {O}}_n^{{cl}_i}}{\partial {\mathscr {V}}_{m,n}^{{cl}_i}} \end{aligned}$$

Equation (9) is obtained from Eq. (8) by applying the chain rule and taking the partial derivative, as shown below.

9 $$\begin{aligned} \Delta {\mathscr {V}}_{m,n}^{{cl}_i} = \text {const} \left( {\mathfrak {a}o}_n^{{cl}_i}-{\mathscr {O}}_n^{{cl}_i} \right) \times {\mathscr {O}}_n^{{cl}_i} \times (1-{\mathscr {O}}_n^{{cl}_i})\times {\mathscr {H}}^{{cl}_i}_m \end{aligned}$$

By substituting the constant factor as provided in Eqs. (9) and (8) can be simplified in Eq. (9). In Eq. (10), $\Delta {\mathscr {V}}_{m,n}^{{cl}_i}$ represents the weight changes between the hidden and output layers, while $\lambda _n^{{cl}_i}$ represents the constant factor described in Eq. (11).

10 $$\begin{aligned}&\Delta {\mathscr {V}}_{m,n}^{{cl}_i} = \text {const} \lambda _n^{{cl}_i} \times {\mathscr {H}}^{{cl}_i}_m \end{aligned}$$

11 $$\begin{aligned}&\lambda _n^{{cl}_i} = \left( {\mathfrak {a}o}_n^{{cl}_i} -{\mathscr {O}}_n^{{cl}_i}\right) \times {\mathscr {O}}_n^{{cl}_i} \times \left( 1-{\mathscr {O}}_n^{{cl}_i}\right) \end{aligned}$$

To update the weights, Eq. (11) can be utilized. In this equation, the next weight ${\mathscr {V}}_{m,n\ }^{{cl}_i}\left( t+1\right)$is modified based on the current weight value ${\mathscr {V}}_{m,n\ }^{{cl}_i}\left( t\right)$, the weight changes $\Delta {\mathscr {V}}_{m,n\ }^{{cl}_i}$, and a learning rate factor $\Upsilon$.

12 $$\begin{aligned} {\mathscr {V}}_{m,n\ }^{{cl}_i}\left( t+1\right) ={\mathscr {V}}_{m,n\ }^{{cl}_i}\left( t\right) +\Upsilon \Delta {\mathscr {V}}_{m,n\ }^{{cl}_i} \end{aligned}$$

By utilizing the chain rule on Eq. (7), we can compute the weight changes from the hidden layer to the input layer. Equation (13) defines $\ \Delta {\mathscr {U}}_{l,m\ }^{{cl}_i}$, representing the change in weights from the $m\mathrm{{th}}$ features of the hidden layer to the ${\ i}\mathrm{^{th}}$ features in the input layer for the ${\ i}\mathrm{^{th}}$ client, ${cl}_i$^47^.

13 $$\begin{aligned} \Delta {\mathscr {U}}_{l,m\ }^{{cl}_i}=-const \frac{\partial {{\mathscr {E}}r}^{{cl}_i}}{\partial {\mathscr {O}}_n^{{cl}_i}} \ \times \frac{{\partial {\mathscr {O}}}_n^{{cl}_i}}{\partial {\mathscr {H}}_m^{{cl}_i}} \times \frac{\partial {\mathscr {H}}_m^{{cl}_i}}{\partial {\mathscr {U}}_{l,m\ }^{{cl}_i}} \end{aligned}$$

Equation (13) can be expressed as Eq. (14) by applying the chain rule and taking the derivative^46,48^.

14 $$\begin{aligned} \Delta {\mathscr {U}}_{l,m\ }^{{cl}_i}=const \left[ \sum _{k}{\left( {\mathfrak {a}o}_n^{{cl}_i} -{\mathscr {O}}_n^{{cl}_i}\right) {\times {\mathscr {O}}}_n^{{cl}_i} \times \left( 1-{\mathscr {O}}_n^{{cl}_i}\right) \times {\mathscr {V}}_{m,n\ }^{{cl}_i}}\ \right]\times {\mathscr {H}}_m^{{cl}_i}\times\left( 1-{\mathscr {H}}_m^{{cl}_i}\right) \times x_i \end{aligned}$$

Equation (14) is simplified to incorporate the constant factor mentioned in Eq. (11).

15 $$\begin{aligned} \Delta {\mathscr {U}}_{l,m\ }^{{cl}_i}=const \left[ \sum _{k}{\lambda _n^{{cl}_i}\times {\mathscr {V}}_{m,n\ }^{{cl}_i}}\right] \times {\mathscr {H}}_m^{{cl}_i}\times \left( 1-{\mathscr {H}}_m^{{cl}_i}\right) \times x_i \end{aligned}$$

Equation (15) can also be simplified to Eq. (16) By substituting the constant factor ${\mathscr {Y}}_m^{{cl}_i}$ shown in Eq. (17).

16 $$\begin{aligned}&\Delta {\mathscr {U}}_{l,m\ }^{{cl}_i}=const{\mathscr {Y}}_m^{{cl}_i} \times x_i \end{aligned}$$

17 $$\begin{aligned}&{\mathscr {Y}}_m^{{cl}_i}=\left[ \sum _{k}{\lambda _n^{{cl}_i} \times {\mathscr {V}}_{m,n\ }^{{cl}_i}}\right] \times {\mathscr {H}}_m^{{cl}_i}\times \left( 1-{\mathscr {H}}_m^{{cl}_i}\right) \end{aligned}$$

The hidden and input layer weights can be updated using Eq. (17), similar to the process described in Eq. (12).

18 $$\begin{aligned} {\mathscr {U}}_{l,m\ }^{{cl}_i}\left( t+1\right) ={\mathscr {U}}_{l,m\ }^{{cl}_i}\left( t\right) +\Upsilon \Delta {\mathscr {U}}_{l,m\ }^{{cl}_i} \end{aligned}$$

The proposed model incorporates Eqs. (12) and (18) for weight updates, considering the learning rate $\Upsilon$. These equations^49,50^ are employed to obtain the optimal weights used for aggregation at the federated server or global model. The pseudocode for the proposed model that executes for the $i\mathrm{^{th}}$ the client is represented in *algorithm*1.

**Algorithm 1:**
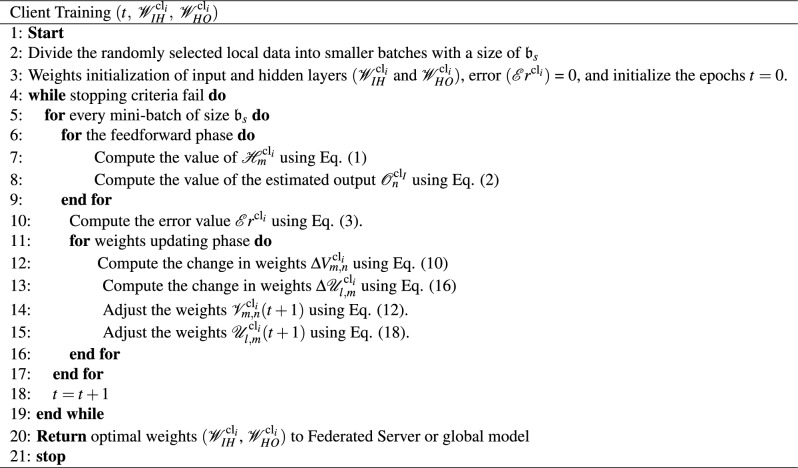
$i\mathrm{^{th}}$ client pseudo code.

The pseudocode in Algorithm 1 outlines the training process for the $i\mathrm{^{th}}$ client in the collaborative model. The client’s training process begins with dividing data into smaller batches with a size of $\mathfrak {b}_s$ for efficient processing. It includes essential phases, including weights initialization for the input and hidden layers $({\mathscr {W}}_{IH}^{\textrm{cl}_i}$ and ${\mathscr {W}}_{HO}^{\textrm{cl}_i})$ respectively, error initialization $({{\mathscr {E}}r}^{\textrm{cl}_i})$ = 0, and the epoch count set to zero ( $t=0$). It proceeds through iterations of mini-batches, employing Eq. (1) for computing hidden layer neuron outputs ${\mathscr {H}}_m^{\textrm{cl}_i}$ and Eq. (2) to estimate the output values ${\mathscr {O}}_n^{\textrm{cl}_i}$ at the output layer during the feedforward phase. The error, computed using Eq. (3), calculates the difference between actual and estimated outputs. The weights updating phase, driven by Eqs. (10) and (16), aims to minimize error by adjusting the weights connecting the hidden and output layers as well as the hidden and input layers. The process continues until a stopping criterion is met, and the optimal weights $({\mathscr {W}}_{IH}^{\textrm{cl}_i}$, ${\mathscr {W}}_{HO}^{\textrm{cl}_i})$ are returned to the federated server, concluding the training process. This pseudocode provides a well-structured and comprehensive representation of the training steps for the $i\mathrm{^{th}}$ client.

### Weights transfer

The weights obtained from Algorithm 1 (whether encrypted or non-encrypted) are transmitted to the global federated server. Encrypting these weights before sending them is possible to enhance the system’s security. However, in this study, the encryption of weights is not implemented, and it is considered an optional component that can be incorporated based on specific application requirements.

### Federated server

Each client transmits its optimal weights ${\mathscr {W}}_{IH}^{\textrm{cl}_i}$, ${\mathscr {W}}_{HO}^{\textrm{cl}_i}$ to the federated server. The aggregated optimal weights for the federated server’s input to the hidden layer can be expressed using Eq. (19), where $w_{IH-FD}^{fl}$ represents the aggregated weights of input to the hidden layer obtained from all locally trained clients.

19 $$\begin{aligned} w_{IH-FD}^{fl}\left( t+1\right) =w_{IH-FD}^{fl} \left( t\right) +\frac{1}{{\mathscr {N}}} \sum _{i=1}^{{\mathscr {N}}}{{\mathscr {W}}_{IH}^{\textrm{cl}_i} \left( t+1\right) } \end{aligned}$$

where $w_{IH-FD}^{fl}\left( t\right)$ refers to the global model parameters at the $t\mathrm{^{th}}$ communication round, while ${\mathscr {W}}_{IH}^{\textrm{cl}_i}$ represents the local model update obtained from the $i\mathrm{^{th}}$ client during communication round $t\ +\ 1$. Similarly, in order to construct the global aggregated model weights for the hidden-to-output layer expressed using Eq. (20), where $w_{HO-FD}^{fl}$ represents the aggregated weights of input to hidden layer obtained from all locally trained clients.

20 $$\begin{aligned} w_{HO-FD}^{fl}\left( t+1\right) =w_{HO-FD}^{fl}\left( t\right) +\frac{1}{{\mathscr {N}}}\sum _{i=1}^{{\mathscr {N}}} {{\mathscr {W}}_{HO}^{\textrm{cl}_i}\left( t+1\right) } \end{aligned}$$

In Eq. (20) $w_{HO-FD}^{fl}\left( t\right)$ refers to the global model parameters at the $t\mathrm{{th}}$ communication round, while ${\mathscr {W}}_{HO}^{\textrm{cl}_i}$ represents the local model update obtained from the $i\mathrm{^{th}}$ client during communication round $t\ +\ 1$. The pseudocode of the steps performed by the federated server is shown in Algorithm 2. It retrieves the weights trained on local models, performs aggregation of these weights, and verifies the validity of the collaborative federated model.

**Algorithm 2:**
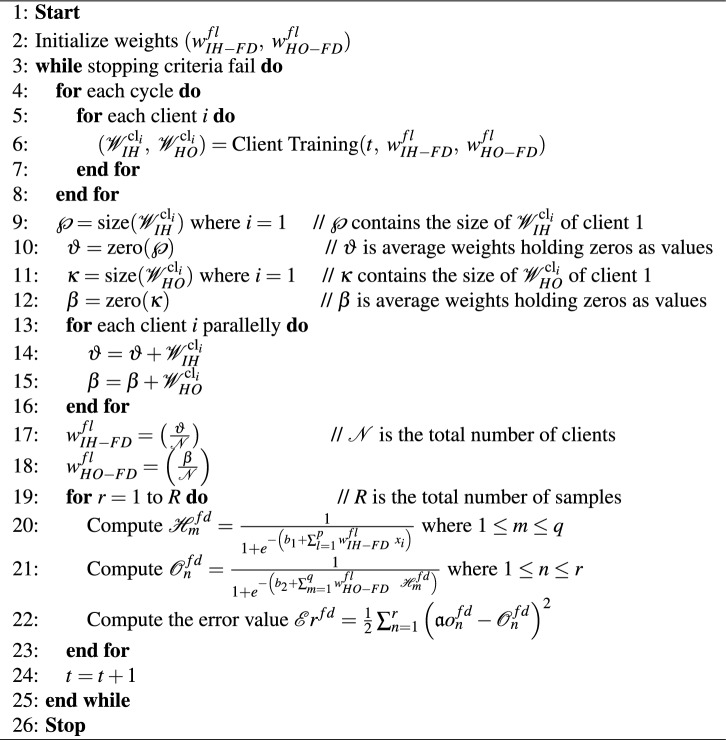
Proposed federated server for the global model.

Algorithm 2 is a pivotal component in the collaborative model and manages the collaboration between the federated server and individual clients. The algorithm initiates with the weight initialization for the model’s input-to-hidden layer ($w_{IH-FD}^{fl}$) and hidden-to-output layer ($w_{HO-FD}^{fl}$) in Step 2. The algorithm then enters a while loop (Step 3) to assess the stopping criteria continually. Within the loop, it iterates through various cycles (Step 4) and clients (Step 5), invoking the ’Client Training’ function to acquire updates from the clients’ local models. Equations (19) and (20) play a pivotal role, where the algorithm processes ${\mathscr {W}}_{IH}^{\textrm{cl}_i}$ and ${\mathscr {W}}_{HO}^{\textrm{cl}_i}$ to update the global model’s input-to-hidden and hidden-to-output layers. Subsequently, the algorithm computes the sizes of these updates ($\wp$ and $\kappa$) and initializes variables $\vartheta$ and $\beta$ to store the average weights, respectively (Steps 9–12). It then processes the individual client updates in parallel (Step 13), adding the weight contributions and finally averaging them over the total number of clients ${\mathscr {N}}$ in Steps 17 and 18. The algorithm further calculates the hidden and output layer values in Steps 20 and 21, which involves the application of the Sigmoid activation function and Eqs. (19) and (20). These values are subsequently used to compute the error in Step 22. This process iterates through a set of samples (*R*) in Step 19 and continues in the while loop until the stopping criteria are met. At each iteration, the global model’s parameters ($w_{IH-FD}^{fl}$ and $w_{HO-FD}^{fl}$) are updated based on the aggregated weights obtained from the clients. This coordinated effort ensures establishing a robust and secure collaborative federated model for effective data analysis and prediction.

### Edge devices

Finally, to enhance the overall safety and security of the ATs network and address passenger demand prediction, the global model will eventually be distributed among regional ATs or network organizations. This collaborative approach improves the accuracy of passenger demand prediction and strengthens the privacy of passenger data. By breaking down the global model, we establish a robust architecture that ensures the safe operation of ATs while safeguarding passenger data privacy and protecting against potential challenges.

## Results and discussion

### Performance evaluation

In ATs demand forecasting, various evaluation metrics are used to measure model accuracy. These metrics include MAE, RMSE and $R^2$. MAE calculates the average absolute difference between predicted and actual demand values, reflecting the average error magnitude. MSE computes the average squared difference, giving more weight to significant errors. RMSE is the square root of MSE, providing an average measure of deviation between predicted and actual demand values. $R^2$ is a statistical measure that indicates how much of the dependent variable’s variation is explained by the independent variables in the model^51^. Higher $R^2$ values indicate a better fit. These metrics comprehensively assess the effectiveness of regression-based approaches for predicting ATs demand. Equations (21) to (26) represents the mathematical form of the above-mentioned evaluation metrics. Where *p* is the total number of samples, ${\mathscr {X}}^{pridicted}$ is the predicted value and ${\mathscr {X}}^{actual}$ is the target value.

21 $$\begin{aligned} MAE&= \frac{1}{p} \sum _{i=1}^{p} \left| {\mathscr {X}}_i^{predicted} - {\mathscr {X}}_i^{actual}\right| \end{aligned}$$

22 $$\begin{aligned} RMSE&= \sqrt{\frac{1}{p} \sum _{i=1}^{p} \left( {\mathscr {X}}_i^{actual} - {\mathscr {X}}_i^{predicted}\right) ^2} \end{aligned}$$

23 $$\begin{aligned} RSS&= \sum _{i=1}^{p} \left( {\mathscr {X}}_i^{actual} - {\mathscr {X}}_i^{predicted}\right) ^2 \end{aligned}$$

24 $$\begin{aligned} TSS&= \sum _{i=1}^{p} \left( {\mathscr {X}}_i^{predicted} - \left( \frac{1}{p}\sum _{i=1}^{p}{\mathscr {X}}_i^{actual} \right) \right) ^2 \end{aligned}$$

25 $$\begin{aligned} R^2&= 1 - \frac{RSS}{TSS} \end{aligned}$$

26 $$\begin{aligned} R&=\sqrt{R^2} \end{aligned}$$

To validate the accuracy of our model, we perform experiments to compare it with several baseline models commonly used for regression-based problems. These models include Random Forest (RF), Support Vector Regression (SVR), Ensemble Bagging (EN-BA), Multi-Layer Perception (MLP), and Gradient Boosting (GB). We use these approaches as benchmarks to evaluate our proposed model. Furthermore, we assess the performance of our approach in comparison to the approaches mentioned in the literature section. RF combines multiple decision trees to capture complex relationships between input features. It provides robust predictions by training these trees on different subsets of the data. We evaluated the effectiveness of RF using five trees to assess its efficiency^52,53^. SVR is a variant of a support vector machine. It is good at handling non-linear and complex datasets by identifying a hyperplane in high-dimensional feature space. It can handle noise and outliers by prioritizing support vectors. In our simulation, we set the epsilon parameter to 0.01, which determines the tolerance margin for errors in the SVR model^54^. EN-BA combines multiple approaches to build a robust model. It uses bagging, which involves creating subsets of training data through random sampling with replacement. This approach is practical for handling noisy or high-dimensional datasets and reduces the risk of overfitting^55^. We utilized an ensemble of 30 decision trees, with a minimum leaf size of 8, for our analysis. In our case, the individual model predictions are averaged to obtain the final output. MLP is another popular approach widely used for taxi demand forecasting, employing interconnected layers of artificial neurons to capture and learn complex non-linear relationships between input features and target variables. It uses backpropagation to train the model by adjusting weights iteratively^56^. We used MLP with ten neurons in each hidden layer. GB combines weak learners (usually decision trees) to form a robust predictive model by capturing complex non-linear relationships. It iteratively trains learners, utilizing residuals from previous iterations to fit new ones^57^. Our experiment used 30 trees with a learning rate of 0.01. Table 3 shows the summary of the baseline models. We carefully selected these settings for each model based on comprehensive research analysis^58–60^ and expert knowledge. They strike a balance between model performance and practical resource constraints, including memory and time considerations.

**Table 3:** Summary of the baseline models.

Model	Description (model settings)
RF	Number of trees: 5, maximum number of features considered for splitting: auto, bootstrap samples: true
SVR	Kernel: gaussian, epsilon: 0.01
EN-BA	Number of decision trees: 30, minimum leaf size: 8, bootstrap samples: true
MLP	Number of hidden layer neurons: 10, hidden layer:2, approach: backpropagation, training epochs: 1000
GB	Number of trees: 30, learning rate: 0.01, maximum depth of trees: 3, maximum number of features considered for splitting: auto, minimum leaf size of 8

This study used the MATLAB R2022b tool to implement the baseline and proposed model and simulate the results. We employed a dataset^44^ containing 7832 samples with four features. The first three features (day name, location, and time slot) uses as inputs to calculate and forecast the fourth feature, which represents the taxi demand for a specific area. Using the same dataset to train and test baseline models ensures a fair evaluation of their performance and enables consistent comparisons. Table 4 presents the performance of the proposed model compared to other baseline approaches. Multiple evaluation metrics, defined in Eqs. (21), (22) and (25), were used to evaluate the proposed model. These metrics measure and compare the performance of the proposed model with the baseline approaches. The analysis shown in Table 4 different approaches and their evaluation metrics reveals distinct performances among the models. The RF approach exhibits moderate results, with an MAE of 5.53 and an RMSE of 15.67, while the SVR approach shows slightly better results, with an MAE of 5.37 and an RMSE of 14.21. However, the EN-BA approach lags with an MAE of 9.92 and an RMSE of 18.53. The MLP approach demonstrates better accuracy, achieving an MAE of 6.19 and an RMSE of 10.69, while the GB approach yields an MAE of 7.66 and an RMSE of 14.09. However, the proposed model stands out among all the approaches with the lowest MAE of 5.32, the lowest RMSE of 9.12, and the highest $R^2$ value of 0.93. This collective performance excellence shows the proposed model’s superiority, solidifying its position as the optimal choice for accurate and precise predictive modelling among the evaluated approaches.

**Table 4:** Comparison of the proposed model with various state-of-the-art approaches.

Metric	RF	SVR	EN-BA	MLP	GB	Proposed model
MAE	5.53	5.37	9.92	6.19	7.66	5.32
RMSE	15.67	14.21	18.53	10.69	14.09	9.12
$R^2$	0.78	0.82	0.69	0.89	0.83	0.93

**Figure 7:**
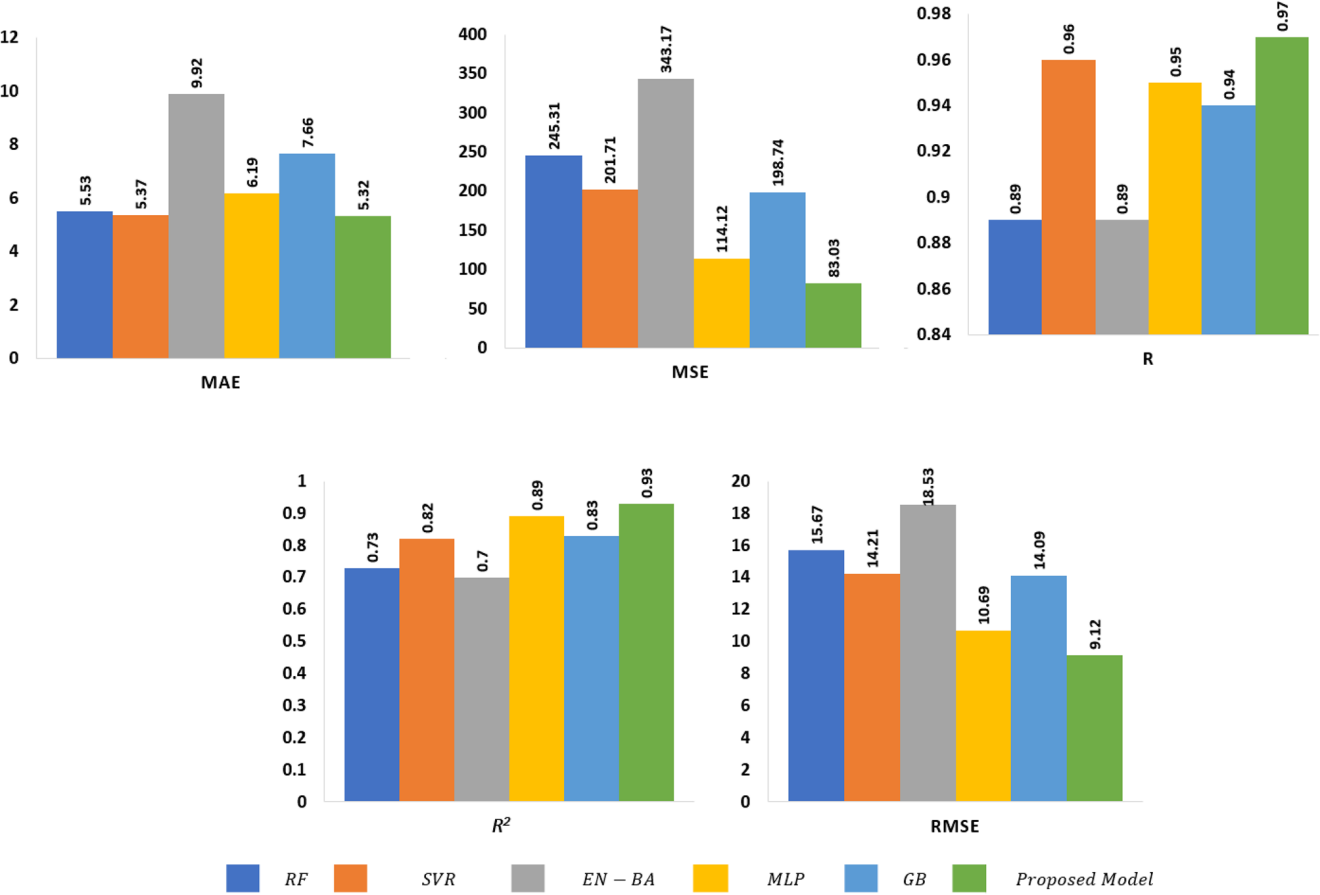
Graphical comparison of the proposed model with various state-of-the-art approaches.

Figure 7 provides a comprehensive graphical illustration of the key performance metrics mentioned in Eqs. (21) to (26). This representation comprises five distinct plots, each illustrating different aspects of the model’s performance. These plots include MAE, MSE, RMSE, R, and $R^2$, comprehensively analyzing the model’s effectiveness. It reveals a consistent trend—the proposed model outperforms baseline models across all metrics. It exhibits the lowest MAE, MSE, and RMSE values, indicating superior precision and accuracy in its predictions. Furthermore, the proposed model consistently exhibits the highest R and $R^2$ values, signifying a superior fit to the data and enhanced explanatory power. In contrast, the baseline models fall short in various aspects, with comparatively higher error metrics and lower explanatory capabilities. These trends highlight the superior performance and predictive accuracy of the proposed model over the other approaches. These findings reassure us that the proposed model strikes a balance between delivering outstanding performance and preserving the privacy of user data.

**Figure 8:**
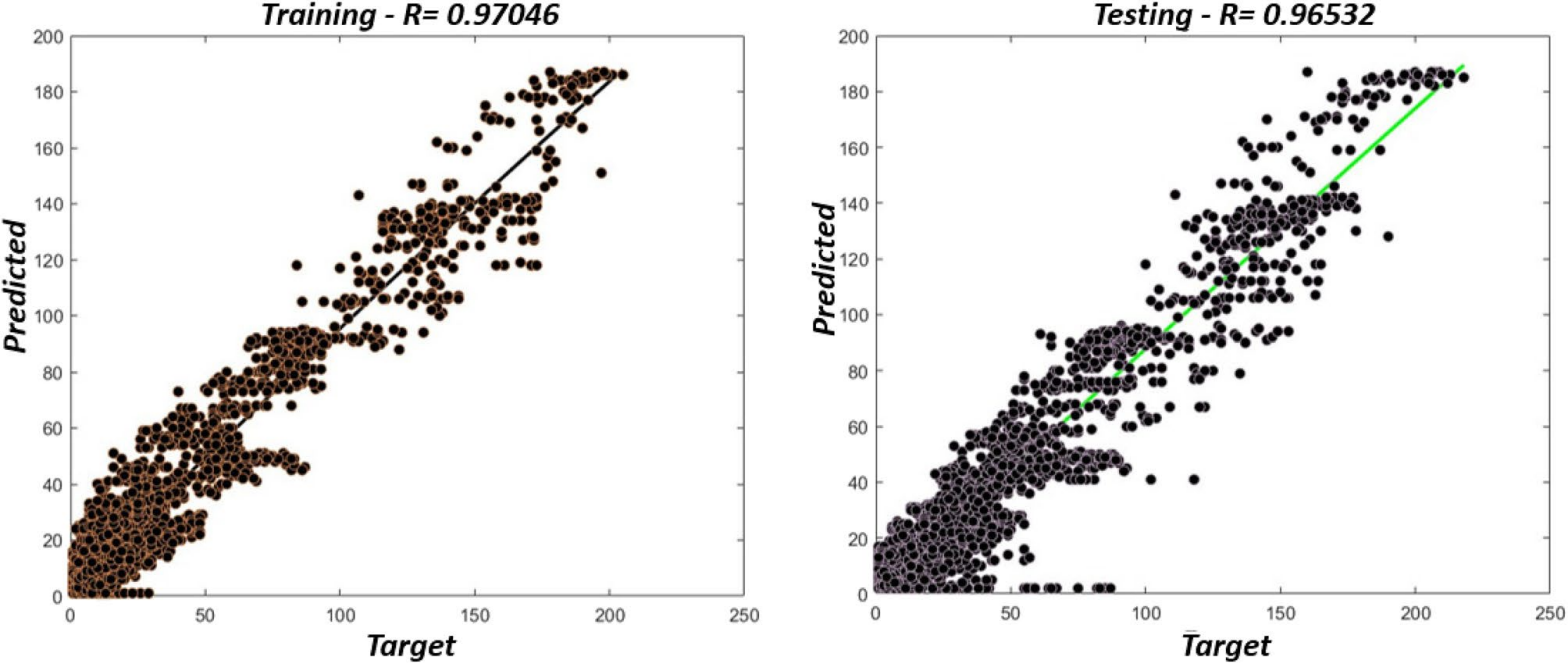
Regression outputs for the training (left) and testing (right) data.

**Figure 9:**
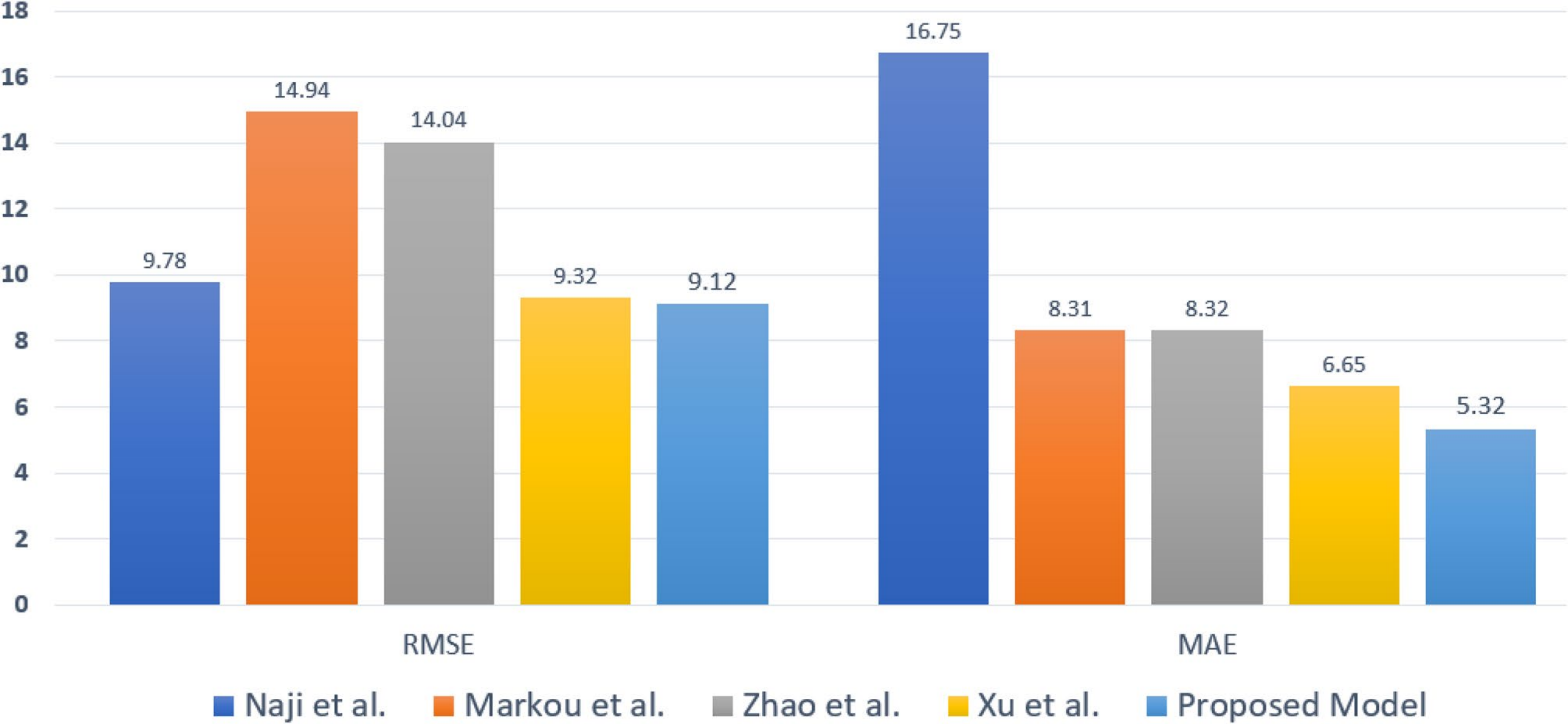
Comparison of the proposed model’s accuracy to previous studies.

The regression outputs for the training and testing data are shown in Fig. 8. In the training data graph, the fit line of the proposed model reveals a robust positive correlation coefficient (R) value of 0.98 by using Eq. (26), indicating a significant relationship between the predicted and actual values. On the other hand, the testing data graph shows an even higher R-value of 0.97, indicating that the proposed model maintains its robust performance and accurately predicts values in the testing data. The proposed model demonstrates superior performance to previous approaches discussed in the literature review section. The previously mentioned models utilized inefficient algorithms that compromise user privacy and collaborative learning, leading to low accuracy and impacting passenger waiting times. It is important to note that higher accuracy reduces passenger waiting times. Passenger demand forecasting is inversely proportional to passenger waiting time, meaning accurate predictions lead to shorter waiting periods. A comparative analysis between the proposed model and earlier approaches described in the literature^25–28^ is presented in Fig. 9, providing a visual representation of the superiority of the proposed model over the existing model. It addresses identified limitations and demonstrates better accuracy in terms of minimum MAE and RMSE.

## Conclusion

This paper introduces a collaborative model based on FL for passenger demand forecasting for ATs within smart cities. The proposed approach enables ATs in different regions to enhance their demand forecasting models through collaboration without directly sharing passenger data. This approach is helpful in training a central model with the help of secure collaboration of different models. To demonstrate the effectiveness of the proposed model, a real-world dataset of over 4500 taxi in Bangkok City is utilized. The results showcase outstanding performance, including the lowest MAE of 5.32, the lowest RMSE of 9.12 and the highest $R^2$ value of 0.93, showing its superiority compared to other approaches. These simulation results emphasize the superior predictive accuracy and overall performance of the proposed model while maintaining user data privacy. This paper suggests exploring an encrypted weight transfer approach to enhance privacy preservation in FL for future research directions. Securely transferring model weights can protect sensitive information, ensuring privacy while facilitating collaborative learning. Furthermore, integrating passenger demand forecasting models with other mobility services enables improved coordination and optimization of urban mobility, such as ride-sharing platforms or public transportation systems, encouraging a holistic approach to transportation management. By sharing demand forecasts across different modes, cities can enhance resource allocation, reduce congestion, and provide efficient and reliable transportation experiences. By considering these future directions, the proposed approach can contribute to advancing passenger demand forecasting for ATs and support the development of more efficient and sustainable urban ITS.

## Data Availability

Publicly available datasets were analyzed in this study. This data is derived from a publicly available dataset provided by the Intelligent Traffic Information Center Foundation, a non-profit organization registered with the Thailand Ministry of Interior. The datasets can be found here: https://itic.longdo.com/opendata/probe-data/.
